# Evaluation of service quality from patients’ viewpoint

**DOI:** 10.1186/s12913-019-3998-0

**Published:** 2019-03-15

**Authors:** Mohammad Ali Abbasi-Moghaddam, Ehsan Zarei, Rafat Bagherzadeh, Hossein Dargahi, Pouria Farrokhi

**Affiliations:** 1 Department of Health Care Management, School of Allied Medical Sciences, Tehran University of Medical Sciences, Tehran, Iran; 2 Department of Health Service Management, School of Management and Medical Education, Shahid Beheshti University of Medical Sciences, Tehran, Iran; 3 School of Health Management and Information Sciences, Iran University of Medical Sciences, Tehran, Iran; 4 Health Information Management Research Center, Tehran University of Medical Sciences, Tehran, Iran

**Keywords:** Patient perception, Service quality, Outpatient services, Quality assessment

## Abstract

**Background:**

Measuring patients’ perception from health service quality as an important element in the assessment of service quality has attracted much attention in recent years. Therefore, this study was conducted to find out how the patients evaluated service quality of clinics at teaching hospitals affiliated with Tehran University of Medical Sciences in Iran.

**Methods:**

This cross-sectional study was conducted in Tehran in 2017 and 400 patients were randomly selected from four hospitals. Data were collected using a questionnaire, the validity and reliability of which were confirmed in previous study. In order to analyze the data, T-test, ANOVA, and Pearson correlation coefficient were calculated using SPSS 23.

**Results:**

The results indicated that among eight dimensions of health service quality, the patients were more satisfied with physician consultation, services costs and admission process. The highest and lowest mean scores were related to physician consultation (Mean = 4.17), and waiting time (Mean = 2.64), in that order. The total mean score of service quality was 3.73 (± 0.51) out of 5. Outpatient services were assessed as good, moderate and weak by 57.5, 40 and 2.5% of the patients, respectively. There was a significant relationship between the positive perception of service quality and reason for admission, source of recommendation, gender, education level, health status, and waiting time in the clinics (*p* <  0.05).

**Conclusion:**

The majority of the patients had a positive experience with visiting clinics and perceived service provision as good. In fact, patients’ perceptions of physician consultation, provision of information to patients and the environment of delivering services, are the most important determinants of service quality in clinics.

## Background

The provision of high quality services is a prerequisite for the success of service organizations since service quality influences patients’ perceived value, their satisfaction and faithfulness [1]; therefore, the improvement of service quality has been on management agenda [2]. Growth in demand for healthcare, increased costs, limited resources, and the variety of clinical interventions have led many health systems in the world to focus on measuring and improving the quality of services. The first step to this end is to define the concept of quality that has long been a topic of much controversy [3, 4].

Service quality is a unique and abstract concept which is difficult to define and measure. Researchers have provided different definitions [5]. It has been described as the judgment or overall attitudes of customers towards the provided services and refers to the differences and mismatches between customers’ expectations and their perceptions of service performance [3, 4]. Quality in health services includes technical (clinical) quality and functional (non-clinical) quality. The former focuses on the skills, accuracy of procedures and medical diagnosis while the latter refers to the way that health services are provided to the patients [6].

Constant monitoring of health services is very important, thus measuring patient perception of health care quality, as a key element in quality assessment, has gained much attention in recent years. Monitoring provides important information about service quality which cannot be obtained through traditional means for performance evaluation [7].

In the past, the process of clinical quality assessment was conducted without considering the viewpoints and feedback of patients; however, nowadays, emphasis is placed on the importance of patients’ views in assessing the quality of services, and mere reliance on clinical effectiveness is not much supported [8]. The feedback and opinions of patients or the voice of clients affect the quality improvement and provides an opportunity for organizational learning [9]. Patients’ perspective of healthcare quality is important for several reasons. First, the high quality of services offered by hospitals is associated with issues, such as patient satisfaction, willingness to re-use services in the future, compliance with doctor’s order, and so on.

Second, patient feedback and perceptions are important requirements for many accreditation and monitoring programs for hospital services. Third, high patient-perceived quality is effectively and positively related to financial performance and profitability of healthcare institutions [10]. Therefore, it can be said that the assessment of service quality helps service providers recognize the specific and often unmet needs of patients and problems in the delivery of services. Moreover, it helps hospital managers design problem-solving and quality-improvement programs [11] and allocate resources more effectively and guarantee high patient satisfaction.

Hospital clinics are one of the most important sources of patients for inpatient departments; consequently, the provision of services in this area affects patients’ overall perception and choice of hospital [12]. Besides, ambulatory (outpatient) care is growing at a faster rate than hospitals, and it is predicted that their revenues would be equivalent or even surpass inpatient revenues in the near future [9]. Most studies in Iran have focused on the quality assessment of primary health care, inpatient service quality and patient satisfaction [13], yet outpatient services have been neglected during the assessment of hospital services. Therefore, this study aimed to answer the following research question: how do the patients assess the quality of services provided by clinics at teaching hospitals affiliated with Tehran University of Medical Sciences?

## Methods

### Study design and sample

This was a cross-sectional study conducted on a random sample of 400 patients who referred to outpatient departments (clinics) in teaching hospitals affiliated with Tehran University of Medical Sciences (TUMS) in Tehran during the first half of 2017. The patients were selected by multistage systematic random sampling, but due to limited time and resources, only four hospitals (two general and two specialized) among 16 were randomly chosen, and each hospital’s share was allocated based on its size (number of beds). Outpatient departments work six days a week, thus in order to increase the likelihood of patient participation in the study, a systematic sampling technique was used to select patients every day from Saturday to Thursday. The patients were then asked to complete a questionnaire before leaving the clinic and following the physician’s consultation. The individual’s consent was a requirement, and the patients who declined to participate in the study (*N* = 14) were substituted by other patients. Since the perception of quality is a subjective judgment, in order to have an accurate yet close-to-reality evaluation, only patients of at least 18 years old and willing to participate were included in the study.

### Instrument

Data were collected using a questionnaire which was designed and validated in a previous study [14]. The reliability of the instrument, in this study, was assessed using Cronbach’s Alpha Coefficient, which ranged from 0.6 to 0.9 for service quality dimensions and 0.92 for overall service quality, indicating the sufficient level of reliability. The questionnaire consisted of two sections; the first part included 13 items on demographic and socio-economic variables, and the second part contained 37 items about hospital’s outpatient services quality; accessibility (three items), appointment (two items), waiting time (two items), admission process (three items), physical environment (six items), physician services (eleven items), disclosure of information to patient (seven items) and cost of services (three items).

The items were measured on a five-point Likert scale ranging from 1 (strongly disagree) to 5 (strongly agree).

### Data analysis

Data were analyzed by SPSS 23 using T-test, ANOVA and Pearson correlation to compare service quality in terms of patients’ demographic variables and assess the relationship between quality dimensions. In addition to the main tests, Friedman and Turkey tests were also used. Regarding the mean score, the overall service quality was divided into three levels; poor (< 2.5), moderate (2. 6–3.75) and good (> 3.75) [14].

## Results

According to the findings, 221 (55.3%) of the patients were male and 290 (72.5%) were married. In terms of education, only 2.3% of the participants were illiterate and most of participants lived in city (86%).Concerning income distribution, the results showed that the majority (63%) of the patients had reported their income as moderate. About 33% of the patients visited hospital clinics once whereas 25% of the patients visited hospital clinics more than 5 times. The results also indicated a postoperative follow-up for 35% of the visits. Most patients (44%) were referred to clinics by their physicians, and the majority of them (about 77%) reported their health status as good or moderate (Table 1). It was also found that the minimum, average and maximum waiting time were 10 min, three, and eight hours, respectively. Furthermore, the lowest, average and the highest service cost were 0.1, 2.25, and 15.5 USD, respectively (Table 2).

**Table 1:** The relationship between demographic characteristics and service quality score (*N* = 400)

Variables	N	%	Mean (±SD)	Test results
Gender				
Male	221	55.3	3.66 (0.56)	T = −2.99 *P* = 0.003
Female	179	44.8	3.81 (0.42)	
Education level				
No schooling	9	2.3	3.04 (0.17)	F = 11.90 *P* < 0.001
Primary and Secondary school	162	40.5	3.82 (0.48)	
University	229	57.3	3.69 (0.51)	
Residence Area				
Urban	344	86	3.72 (0.51)	T = 0.13 *P* = 0.89
Rural	56	14	3.73 (0.47)	
Marital status				
Married	290	72.5	3.70 (0.54)	F = 1.71 *P* = 0.14
Single	88	22	3.78 (0.52)	
Widowed	10	2.5	4.04 (0.15)	
Divorced	12	3	3.64 (0.19)	
Economic status				
Excellent	2	0.5	4.14 (0.01)	F = 1.46 *P* = 0.22
Good	62	15.5	3.80 (0.56)	
Average	250	62.5	3.69 (0.50)	
Low	86	21.5	3.77 (0.50)	
Rate of clinic visit				
First	130	32.5	3.70 (0.51)	F = 3.52 *P* = 0.08
Second	78	19.5	3.81 (0.54)	
Third	60	15	3.78 (0.44)	
Fourth	34	8.5	3.91 (0.45)	
Fifth or more	98	24.5	3.59 (0.51)	
Reason for admission				
New disease	136	34	3.81 (0.49)	F = 5.50 *P* = 0.04
Postoperative follow-up	139	34.8	3.62 (0.49)	
Previous disease	125	31.2	3.75 (0.53)	
Source of recommendation				
Doctors	176	44	3.76 (0.55)	F = 2.33 *P* = 0.04
Family	66	16.5	3.63 (0.36)	
Friends or Relatives	110	27.5	3.69 (0.52)	
Media	15	6.3	3.89 (0.50)	
Other patients	23	5.8	3.73 (0.43)	
Health status				
Excellent	27	6.8	3.89 (0.46)	F = 2.67 *P* = 0.04
Good	125	31.3	3.75 (0.54)	
Fair	182	45.5	3.73 (0.51)	
poor	66	16.5	3.59 (0.43)

**Table 2:** The Relationship between age, waiting time and patient payment with service quality score

	Mean	SD	Correlation coefficient	*p*-value
Age	39.9	14.4	−0.017	0.730
Waiting time (min.)	185	99	−0.469	< 0.001
Out of pocket payment (USD)	2	2	−0.090	0.072

The findings on service quality dimensions indicated the highest mean score related to physician’s consultation (4.17) and the lowest to patient waiting time (2.64). Service quality dimensions, according to Friedman’s test, were ranked as follows; physician’s consultation, perceived service costs, admission process, disclosure of information to patient, physical environment, appointment, accessibility and perceived waiting time (Table 3).

**Table 3:** Mean and standard deviations of service quality dimensions

Dimensions	Mean	SD	Min score	Max score	Average rating (Friedman test)
Accessibility	3.23	0.82	1	5	4.06
Appointment	3.32	1.18	1	5	4.79
Waiting time	2.64	1	1	5	2.57
Admission process	3.94	0.76	2	5	6.89
Physical environment	3.33	0.78	1	5	4.16
Physician’s consultation	4.17	0.60	2.55	5	7.84
Information provision to patient	3.74	0.83	1.43	5	5.71
Service costs	4.15	0.84	1	5	7.79
Service quality	3.73	0.51	2.24	5	5.55

Based on the findings, 2.5% of the respondents assessed the quality of outpatient services as poor, 40% as moderate and 57.5% as good. Concerning to service quality dimensions, the patients were mostly satisfied with physician’s consultation (78.3%), cost of the services (76.5%) and admission process (62.5%). The patients were least satisfied with waiting time which was evaluated as poor by 58% of the patients (Table 4).

**Table 4:** Clinics service quality status from patient’s perspective

Dimensions	Good		Moderate		Poor	
	N	%	N	%	N	%
Accessibility	93	23.3	226	56.5	81	20.3
Waiting time	67	16.8	101	25.3	232	58
Admission process	250	62.5	128	32	22	5.5
Physical environment	125	31.3	209	52.3	66	16.5
Physician’s consultation	313	78.3	87	21.8	–	–
Information provision to patient	221	55.3	146	36.5	33	8.3
Service costs	306	76.5	74	18.5	20	5
Appointment	190	47.5	97	24.3	113	28.3
Service quality (Total)	230	57.5	160	40	10	2.5

Furthermore, a significant correlation was found between overall service quality and its dimensions, specifically physician’s consultation (*r* = 0.766) which was followed by other dimensions, such as providing information to patient, physical environment, accessibility, appointment, perceived service costs and waiting time (Table 5).

**Table 5:** Correlation between service quality and its dimensions

	Information provision to patient	Physician’s consultation	Admission process	Accessibility	Appointment	Waiting time	Physical environment	Service costs	Service quality
Information provision to patient	1								
Physician’s consultation	0.584	1							
Admission process	0.163	0.234	1						
Accessibility	0.264	0.309	0.336	1					
Appointment	0.199	0.176	0.289	0.465	1				
Waiting time	0.225	0.271	0.334	0.317	0.331	1			
Physical environment	0.313	0.349	0.274	0.343	0.410	0.445	1		
Service costs	0.275	0.231	0.346	0.318	0.301	0.219	0.377	1	
Service quality	0.729	0.766	0.520	0.579	0.557	0.533	0.693	0.537	1

All correlation was significant at the 0.01 level (2-tailed)

Comparison of mean scores of service quality in terms of demographic variables showed that the highest quality score was achieved by female patients, the patients who referred to clinics due to new disease and those who were familiar with clinics through media. Service quality was improved by increasing education level and health status and reducing waiting time at clinics. No significant relationship was found between other variables and service quality score (Tables 1 and 3).

## Discussion

This study aimed to evaluate clinics service quality of teaching hospitals in Iran from the patients’ viewpoint and results showed that the overall services quality was assessed as good by 57.5% of the patients while 2.5% of the patients defined it as poor. The findings of the study indicated a better status of service quality compared with the service quality in Shiraz teaching hospitals clinics where about 37% of the patients were satisfied with service quality [12]. In a study conducted by Mpinganjira, the patients reported status of service quality as good [8]. In another study at cancer clinics in Canada [15], the quality score was reported above average (3.66) which is consistent with our result.

The findings demonstrated that the highest score of service quality was attributed to the physician’s consultation. Patients often lack sufficient information and knowledge to assess the medical staff, and perhaps this is the reason why they tend to assess them positively [16]. It should also be noted that in the process of health service delivery, patients are more sensitive to care provided by physicians and nurses [17, 18]; in fact, human elements are more important compared with non-human elements in patient perception of care quality [19]. Doctor-patient interpersonal relationship also plays a key role in shaping service quality judgments [20]. Personal relationships greatly affect the service quality perception since the services are intangible and inseparable from consumers [21]. The findings of studies in Greece, Norway, France and Finland, also indicated that the highest mean score was related to the quality of physician’s consultation [22–25].

Service costs and admission process ranked as the second and third highest dimensions of outpatient services quality. A study in Iran also showed that patients were satisfied with the cost of outpatient services which is similar to our findings [14]. According to the health insurance law in Iran, the amount of patient copayment for outpatient services is 30% of the services cost [26] and in public hospitals, outpatient services such as physician’s consultation are fully covered by health insurance plans. Therefore, patients pay a small amount for the outpatient services and are expected to be satisfied with this dimension of service quality.

The provision of information to patients which had a high correlation with service quality, took the fourth rank in this study. This is in contrast with the findings of other studies in which the patients did not give a high score to the quality of information; consequently, this dimension was not included in the highest ranked dimensions [8, 14, 16, 27].

The appointment process, which ranked fifth, was perceived as moderate and good by approximately 72% of the patients. The negative perception could be attributed to bureaucratic processes, lack of proper appointment systems, or inappropriate staff behavior. The results are in line with those the findings of studies conducted in Greece and Norway where the patients also perceived the quality of appointment process as good and moderate [16, 22, 23].

The sixth rank was related to the clinic environment where the most important reason for dissatisfaction seemed to be due to poor hygiene and insufficient numbers of seats. This is in accord with the findings of other studies in which the quality of facilities and physical environment ranked four among five items [28, 29]. Although the quality of clinic environment does not stand in a good position in the overall ranking, the majority of the patients had positively perceived it as moderate and good (about 83%). This is also in line with the findings of a study in Johannesburg private clinics, South Africa [8] as well as a study in outpatient cancer clinics in Canada [15] where the patients had a positive perception of the physical environment.

The least positive perception of service quality was related to waiting time and accessibility to outpatient services. Long waiting time is the most important reason for dissatisfaction and decreases patients’ positive perception of services quality [30]. Previous studies have also indicated that long waiting time at the clinic and inaccessibility to hospital outpatient services, affect patients’ dissatisfaction with service quality [25]. It has also been found that patients had the least positive perception of waiting time for visiting the physician [14, 15, 31].

Furthermore, the results indicated a significant relationship between gender, education level, reason for admission, source of recommendation, health status and waiting time in the clinic, and service quality. In this study, unlike the previous studies, the male patients had higher expectations compared with the female patients and were dissatisfied with service quality [6, 32]. There was a statistically significant difference between the patients’ perceptions of quality and their education, meaning that less educated patients had the least positive perception of service quality. It seems that lower education leads to more illogical expectations, and this is in contrast with the results of other studies [6].

Those patients, who referred to clinics due to new health problems, had a more positive perception in comparison with the other patients. This could be attributed to some factors, such as recovery from their previous illnesses, hoping for recovery in the selected clinic, or lack of familiarity with the details and shortcomings in the service delivery processes. The findings showed that the patients who got familiar with clinics through media, gave higher scores to service quality, this could be due to the fact they might have received the same services. It was also found that the patients with better health status had lower expectations and more positive perceptions. This was consistent with other studies in which health status was confirmed to be one of the determinants of patient satisfaction with service quality [6, 24, 32, 33].

There was no significant relationship between service costs and age with service quality; however, they were negatively correlated with the perception of service quality, meaning that higher cost and older age led to less positive perception of quality. Waiting time in clinics had a significant inverse relationship with the perception of service quality which has been expected. It means that long waiting time was associated with lower positive perceptions of service quality. The same relationship was found in other studies [12, 31].

Delays in the provision of hospital services are one of the key issues in care quality and can lead to a negative perception of the provided service quality if considered as unreasonable and unnecessary by patients [34]. Therefore, hospitals should design patient-oriented service processes rather than personnel-oriented and improve quality of service delivery through education and system design [35].

### Study limitations

As any other study, this research has some limitations. Healthcare quality is a broad concept that is affected by several factors and cannot be adequately explored through quantitative studies. However, a triangulation of key informant interviews and focus group discussion with patients and service providers would provide more insight into this area. Therefore, it is suggested that potential researchers use the triangulation design to assess the quality of services.

## Conclusions

According to the findings, the majority of the patients had a positive experience with visiting clinics at teaching hospitals and perceived the service quality as good (approximately 58%). The most positive perceptions of the patients were related to the quality of physician consultation, service costs, admission processes, and information provision to patient. Also, physician consultation and providing information to patient were two factors determining clinic’s service quality. For that reason, it is suggested to improve the ‘disclosure of information to patients’ which is one of the most important factors in service quality, and use web based appointment system to reduce waiting time for physician appointment. It is also recommended that clinics improve their physical environment to increase their patient’s positive perceptions. The findings could be valuable for healthcare managers/providers and provide them with useful information about the special needs of their patients and the existing problems. In this case, they can channel their efforts to satisfy their patients’ demands and eliminate the weak points.

## Abbreviations

ANOVA: Analysis of variance
TUMS: Tehran University of Medical Sciences
